# Sexual functioning in patients with cancer

**DOI:** 10.1192/j.eurpsy.2022.2080

**Published:** 2022-09-01

**Authors:** A. Guermazi, S. Hentati, R. Masmoudi, F. Guermazi, I. Baati, I. Feki, J. Masmoudi

**Affiliations:** Hedi Chaker University Hospital, Sfax, Tunisia, Department Of Psychiatry A, Sfax, Tunisia

**Keywords:** Female sexual Function Index, International Index of Erectile Function, cancer, Sexual functioning

## Abstract

**Introduction:**

Sexuality is a growing field in the context of the management of chronic diseases and cancer in particular. Cancer treatments and the traumatic nature of the cancer experience frequently elicit considerable sexual difficulties.

**Objectives:**

To assess the prevalence of sexual dysfunction (SD) in patients with cancer, and to determine the associated factors.

**Methods:**

This was a cross-sectional study, conducted over 1 month, involving 100 cancer patients followed in the oncology department at the Habib Bourguiba University Hospital in Sfax (Tunisia). General, clinical and therapeutic data were collected using a pre-established questionnaire. Sexual function was assessed with the “Female sexual Function Index” and the “International Index of Erectile Function”.

**Results:**

These results showed that half of the patients were female, and 70% of them were married. Their mean age was 51.96, and 68% of them were unemployed. Unemployment in men and treatment with chemotherapy were statistically associated with erectile dysfunction (p=0.049 and p= 0.001 respectively). treatment with radiotherapy was statistically associated with decreased desire in men (p=0.048). Depression correlated with a decreased orgasm (p=0.032) and erectile dysfunction (p=0.043) in men, mean score of IIEF (p= 0.019) and with a decreased sexual arousal (p=0.006) in women.

**Conclusions:**

Sexual dysfunction is common in cancer patients. They can be of iatrogenic or psychological origin and can depend on the dynamics of the couple relationship. Training to raise awareness of the importance of sexuality first among cancer patients should be considered given the lack of communication between doctors and patients regarding sexuality issues.

**Disclosure:**

No significant relationships.

